# Probing the Transfer of the Exchange Bias Effect by Polarized Neutron Reflectometry

**DOI:** 10.1038/s41598-019-43251-1

**Published:** 2019-04-30

**Authors:** X. Z. Zhan, G. Li, J. W. Cai, T. Zhu, J. F. K. Cooper, C. J. Kinane, S. Langridge

**Affiliations:** 1 Beijing National Laboratory for Condensed Matter Physics and Institute of Physics, Chinese Academy of Sciences, Beijing, 100190 China; 2 Dongguan Neutron Science Center, Dongguan, 523803 China; 3 ISIS Neutron and Muon Source, Rutherford Appleton Laboratory, Harwell Campus, Didcot, OX11 0QX UK; 4 Songshan Lake Materials Laboratory, Dongguan, Guangdong, 523808 China

**Keywords:** Magnetic properties and materials, Spintronics

## Abstract

The magnetic reversal behavior of a ferromagnet (FM) coupled through an FeMn antiferromagnet (AF) to a pinned ferromagnet has been investigated by polarized neutron reflectivity measurements. With FeMn as the AF layer it is found that there exists 90° interlayer coupling through this layer and that this plays a key role in the transfer of the exchange bias (EB) effect from the FM/AF interface to the AF/pinned-FM interface. Combined with Monte Carlo simulations, we demonstrate that the competition between the interlayer coupling and the anisotropy of the AF layer results in a control of the EB effect which has potential for device applications.

## Introduction

Exchange bias (EB) manifests itself as a shift of the hysteresis loop along the cooling field direction for a ferromagnet (FM) exchanged coupled to an antiferromagnet (AF). After its discovery by Meiklejohn and Bean in oxidized cobalt particles^1^ the myriad of applications have inspired an enormous amount of research. Many early reports focused on the AF interface spins which are believed to play a key role in achieving an EB effect^2–4^. On the other hand, AF ordered spins in the bulk of the AF layer are also considered to be very important for the EB effect^5–9^. To fully understand the significance of the AF spin structure a FM/AF/FM trilayer has become an ideal structure for investigations^8,10–16^. 90° interlayer exchange coupling between the FM layers has been found through a metallic AF layer, such as Mn^17^ and Cr^18^, and even through an insulating AF layer, such as NiO^19,20^. The spin structure in the AF bulk during the FM reversal is still not fully resolved and an understanding of the characteristics of the AF behavior is not only of fundamental interest, but also for the exploitation of EB in spintronic applications^21–23^. AF materials have become more and more important in modern spintronic applications by progressing from providing pinning for FM layers through to significant transport effects^21,24^. This is further complemented by their robustness against magnetic field perturbations, and high operation frequency^22^. Furthermore, due to the 90° interlayer coupling through NiO as also occurs in YIG/NiO/Pt^25,26^, a novel long-range spin transport application can be imagined^26,27^.

If the bulk (away from the interface) characteristics of the AF are important, this would imply that EB in FM/AF/FM trilayers could propagate from one AF/FM interface to the other^28^. In our previous measurements^29–31^, we have studied the magnetic reversal behavior in NiFe1/FeMn/pinned-NiFe2 multilayers, in which the bias field at the NiFe1/FeMn interface is dependent on the magnetic reversal measurement loop and which displayed an obvious difference in the minor and major hysteresis loops of the NiFe1 layer^30^, suggesting a transfer of the EB effect. For a thin FeMn layer (1 nm), in which the blocking temperature of FeMn is well below room temperature, a 90° interlayer coupling has been observed through polarized neutron reflectivity (PNR)^31^. However, the interlayer coupling across the bulk FeMn and its effect on the transfer of EB remain unclear. Indeed, the mechanism of 90° interlayer coupling in the FM/NiO/FM trilayers with thick NiO thickness is also not well understood^19,20,32^. In this paper, we investigate the EB effect in NiFe1/FeMn/pinned-NiFe2 multilayers for thick FeMn spacer layers (above a critical AF thickness *t*_c_), which possesses a strong enough intrinsic anisotropy to stand against the spin fluctuation (induced by thermal effect or interface roughness) and to pin a FM layer at room temperature^30^. Here, *t*_c_ is the AF thickness, and the EB field at room temperature vanishes when the thickness of the AF is smaller than *t*_c_. Based on the PNR results, 90° interlayer coupling through the thick FeMn spacer layer has been identified and allows a direct observation of the changes of the coupling angle between both the FMs with increasing AF thickness. Our experimental results are confirmed by detailed Monte Carlo simulations, and we demonstrate that the competition between 90° interlayer coupling and the AF anisotropy results in the transfer of the EB effect across the thick AF layer.

## Experiment

**Figure 1:**
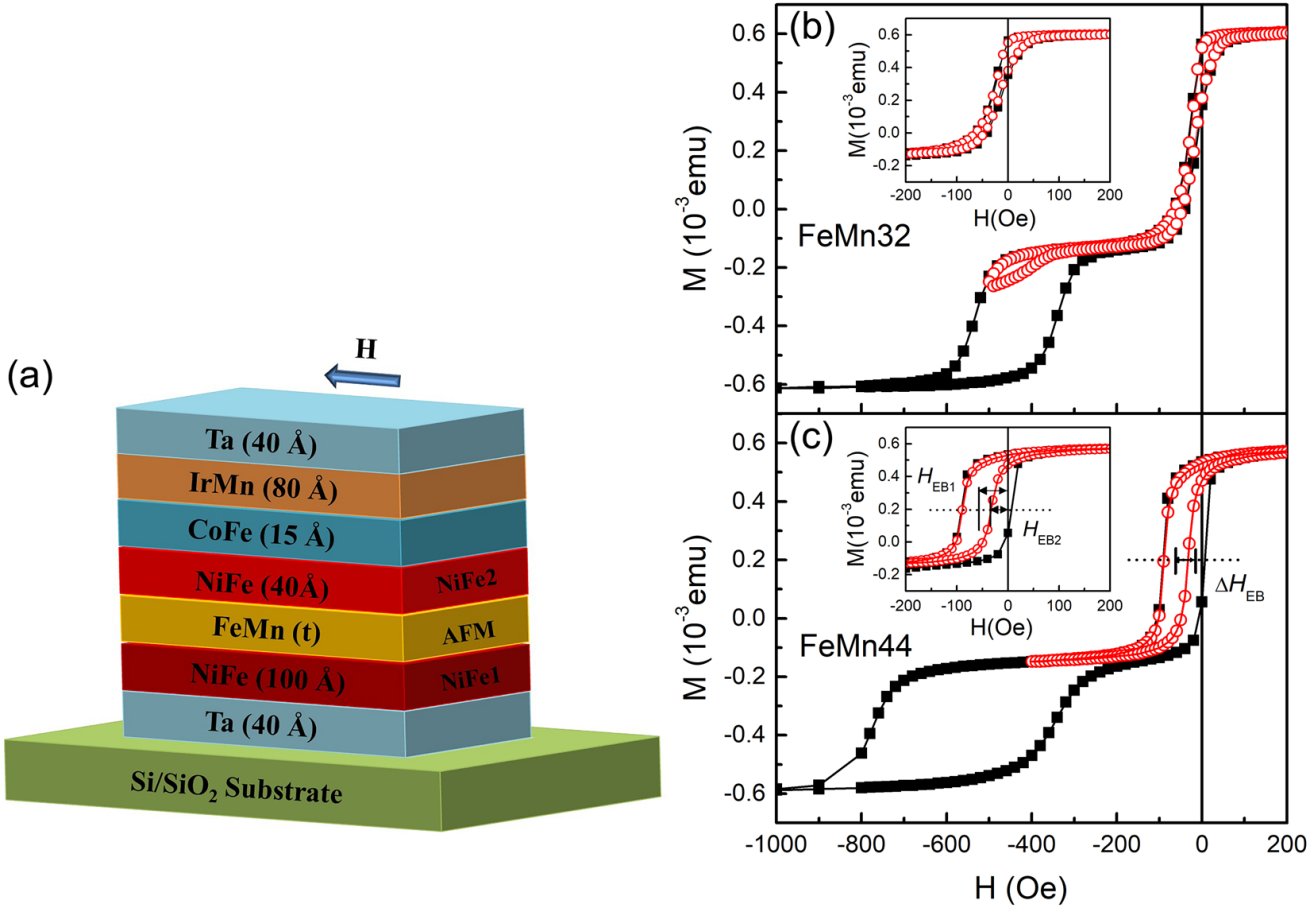
(**a**) The NiFe1/FeMn/pinned-NiFe2 multilayer structure with the numbers in the parentheses indicating the thickness of each layer. The arrow indicates the direction of the deposition field, the cooling field and the external field during measurements which are parallel to each other. The representative hysteresis loops recorded by VSM at room temperature for the FeMn32 sample (**b**) and FeMn44 sample (**c**). The total magnetization of the thin film structure is represented as *M*. The insets show the enlarged NiFe1 hysteresis loops. Red circles represent the minor loop, while the black squares represent the major loop. *H*_EB1_ and *H*_EB2_ represent the EB fields of NiFe1 layer for the minor loop and the major loop respectively. The difference between both EB fields is denoted by ∆*H*_EB_ = *H*_EB1_− *H*_EB2_.

The samples were grown on thermal oxidized Si wafers in a sputtering chamber with a base pressure of 3 × 10^−5^ Pa. The Argon pressure for sputtering is 0.6 Pa. As shown in Fig. 1(a), the samples consist of a Ta(40)/Ni_81_Fe_19_(100)/Fe_50_Mn_50_(*t*_FeMn_)/Ni_81_Fe_19_(40)/Co_50_Fe_50_(15)/Ir_25_Mn_75_(80)/Ta(40) stacking sequence with *t*_FeMn_ ranging from 32 to 70 Å. The numbers in brackets are nominal thicknesses in Ångstrom. The Ta buffer layer at the bottom promoted a (111) texture during the deposition of the multilayers and the Ta capping layer at the top protects against oxidation^29^. The Co_50_Fe_50_/Ir_25_Mn_75_ layers functioned as pinning layers to bias the top NiFe layer (NiFe2). For simplification, we shall hereon refer to a trilayer-like system as NiFe1/FeMn/pinned-NiFe2. The films were magnetically characterized using a vibrating sample magnetometer (VSM).

PNR measurements were performed on the Offspec and PolRef reflectometers at the ISIS neutron source of the Rutherford Appleton Laboratory^33^, and also on the multipurpose reflectometer (MR) at the China Spallation Neutron Source (CSNS)^34^. PNR is a powerful tool for determining the absolute magnitude and orientation of the magnetic induction in thin films and allows us to probe buried interfaces in magnetic multilayers^35,36^. By polarizing the incoming neutron spin eigenstate and analyzing the reflected spin state, two types of neutron reflectivity can be obtained, *i.e*. the non-spin-flip (NSF) and the spin-flip (SF) reflectivities. For NSF neutron reflectivities (R^++^ and R^−^), the spin polarizations are the same for the incoming and reflected neutrons, and the component of the magnetization parallel or antiparallel to the neutron quantization direction can be determined. As for the SF neutron reflectivity (R^+−^ and R^−+^), the reflected neutrons possess an opposite spin polarization with respect to the incoming neutrons. The component of magnetization perpendicular to the incident neutron quantization direction can thus be probed. Combining the results from NSF and SF neutron reflectivities allows the magnitude and the direction of the magnetic induction to be determined *i.e*. a quantitative depth dependent vector magnetometer.

## Exchange Bias Effect

Figure 1(b,c) show the representative magnetic hysteresis loops of the samples with FeMn thickness of 32 Å and 44 Å. These samples are named FeMn32 and, FeMn44 respectively hereafter. Since the loops are well separated in field we can perform two types of hysteresis loops: a major loop, where both magnetizations of NiFe1 and pinned-NiFe2 layers reverse with the external field, or a minor loop, where only the NiFe1 layer reverses with the external field. The EB fields of NiFe1 layer for the minor loop (*H*_EB1_), the major loop (*H*_EB2_), and the EB field difference (∆*H*_EB_ = *H*_EB1_− *H*_EB2_) can be determined. As depicted in Fig. 1(b) the minor loop and major loop for the NiFe1 layer in FeMn32 sample fall on top of each other and show almost the same EB fields (*H*_EB1_ = 22.9 Oe, *H*_EB2_ = 22.4 Oe). The linear and tilted hysteresis sub-loop of the NiFe1 layer can be observed in Fig. 1(b), and indicate that the magnetic measurement field is along the hard axis of the NiFe1 layer. However, the sub-loop of the pinned NiFe2 layer is almost square since the magnetic measurement field is along its easy axis. These features confirm the 90° interlayer coupling between both NiFe layers^29^. With increasing FeMn thickness, the hysteresis loops of the NiFe1 layer in FeMn44 sample become much squarer in shape. As shown in Fig. 1(c), the EB fields of the NiFe1 layer in both hysteresis loops (*H*_EB1_ = 60.2 Oe and *H*_EB2_ = 41 Oe) become significantly larger compared to the FeMn32 sample. To explain the EB, Mauri *et al*. employed a domain wall into the AF with a uniaxial anisotropy^37^, *K*_AF_, which gradually increases with the increase in the AF thickness^38–40^. The increase of the observed EB field we ascribe to an increase in the AF anisotropy with thickness. We shall return to this increase in the discussion of the Monte Carlo simulations. Moreover, an obvious transfer of the EB effect, *i.e*., the difference between the major loop and minor loop of NiFe1 layer (∆*H*_EB_ = 19.2 Oe), can be seen for the FeMn44 sample.

**Figure 2:**
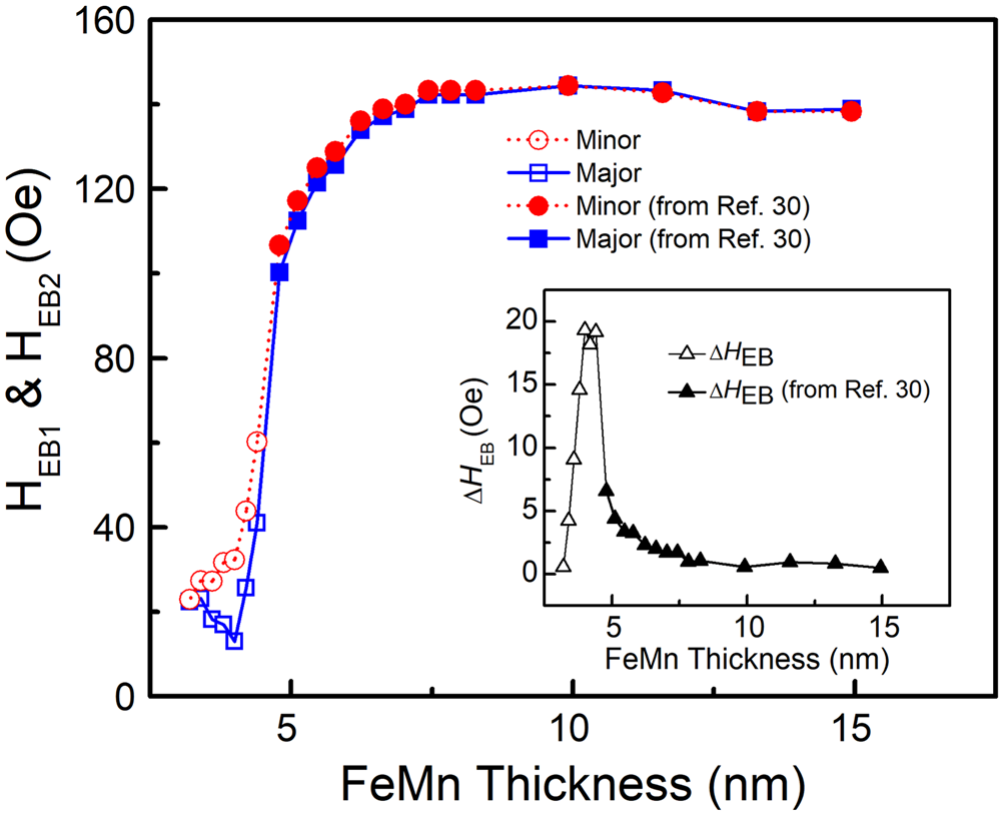
The *t*_FeMn_ dependence of EB fields for the minor loop *H*_EB1_ (circles) and the major loop *H*_EB2_ (squares). The inset plots the EB field difference ∆H_EB_ (triangles) as a function of *t*_FeMn_. The open symbols represent the measured results from our samples, while the solid symbols indicate data extracted from ref.^30^.

In Fig. 2, we show the EB fields of NiFe1 (*H*_EB1_ and *H*_EB2_) as a function of *t*_FeMn_. Both *H*_EB1_ and *H*_EB2_ increase with increasing *t*_FeMn_. As shown in the inset of Fig. 2, the EB field difference Δ*H*_EB_ experiences a rapid enhancement when *t*_FeMn_ > 32 Å and reaches a maximum of 19.3 Oe when *t*_FeMn_ is about 40 Å. The EB field vanishes at room temperature when the thickness of the AF is smaller than a critical thickness. In our experiments, the critical thickness of FeMn is about 24 Å (see the supplementary materials). Similar with our previous report^30^, the EB field difference disappeared when *t*_FeMn_ > 70 Å, where the minor and major loops are both square and again overlap with each other. This result suggests that the investigation of the EB transfer across the AF spacer layer, which is well above the AF critical thickness, could provide further insights into its underlying physical mechanism.

## Polarized Neutron Reflectivity

**Figure 3:**
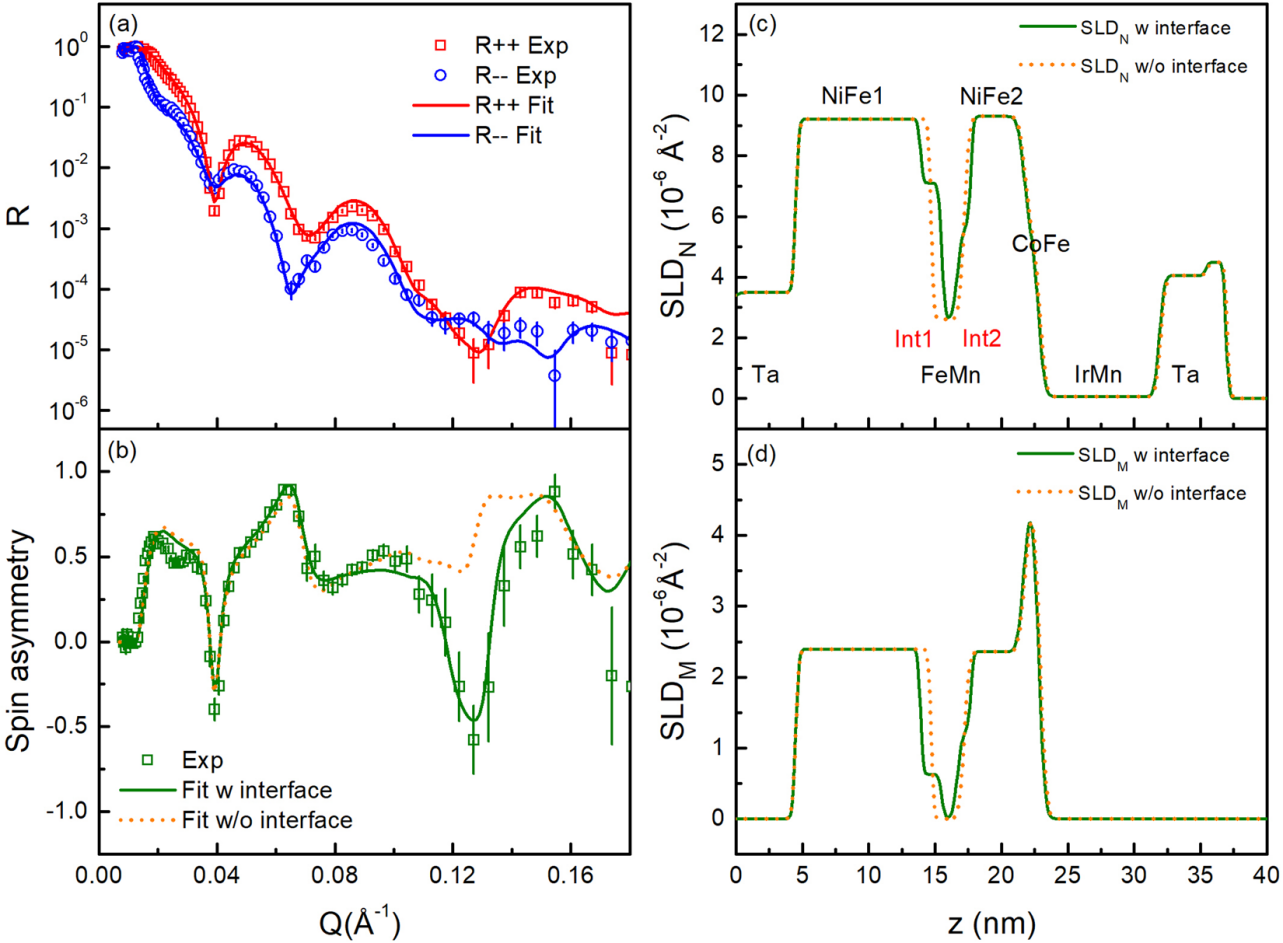
(**a**) The NSF PNR reflectivities for the FeMn32 sample in the saturation state. (**b**) The PNR spin-asymmetry ratio obtained from the experimental and the calculated reflectivities for thin film models with interfacial layers (green solid line) and without interfacial layers (orange dashed line). (**c**) The nuclear SLD_N_ and (**d**) magnetization SLD_M_ profiles for fitting models with interfacial layers (solid line) and without interfacial layers (dashed line).

PNR measurements were first performed at a saturation field 0.7 T. At saturation the SF reflectivities are zero (*R*^+−^, *R*^−+^ = 0) and we can simply analyze the NSF reflectivity. By fitting the NSF reflectivity curves with the SimulReflec software^41^, the saturation magnetization profile and the scattering length density (SLD) profiles were obtained. Figure 3 shows the PNR data and fitting results for the FeMn32 sample. We found that to obtain a good agreement between our simulation and the experimental data for all wave-vector transfers *Q* we had to add two interfacial layers at the NiFe/FeMn interfaces as shown in Fig. 3(c), which we call Int1 and Int2. To understand these interlayers, we compare two kinds of layer structures. One in which there are interfacial layers between the NiFe and FeMn layers and a second without such interfacial layers. For the latter case, the fitting results of both the nuclear SLD profile and the magnetization SLD profile show sharp transitions at the FeMn/NiFe interfaces (dashed curves in Fig. 3(c,d)). The spin-asymmetry (SA), defined as SA = (*R*^++^−*R*^−−^)/(*R*^++^+*R*^−−^)is a more magnetically sensitive measure of the reflectivity. We obtain the experimental SA and show the simulated SA as a function of scattering vector *Q*, in Fig. 3(b) for the two-layer models described. It is clear that the simulated SA from the layer model with interfacial layers agrees better than the model with no interface layers for the entire *Q* range. This is true for all of the FeMn thicknesses measured. This model of interfacial layers agrees with previous reports of uncompensated spins at the FM/AF interfaces by PNR measurements^42–45^, and by x-ray techniques^46,47^ in a variety of systems, such as Co/FeF_2_^42^, Py/CoO^46^, NiFe/FeMn^47^. From our subsequent analysis we see that these uncompensated moments are not frozen and are able to rotate with applied field. Meanwhile, significant interdiffusion at interfaces has been widely reported in NiFe/FeMn systems^40,48,49^. In addition, the sputtering sequence has also been shown to influence the interface mixing^49,50^. The sputtered NiFe/FeMn interface can show a more significant interdiffusion than the FeMn/NiFe interface. Therefore, compared to the FeMn/NiFe interface, the interfacial layer at the NiFe/FeMn interface should be thicker due to greater interface mixing and also to have a weaker magnetization. Both points agree well with our observation that the Int1 layer is thicker and with a smaller magnetic scattering length density (SLD_M_) whilst the Int2 region is thinner with a larger SLD_M_ as shown in Fig. 3(d). All the analysis presented hereafter is based on the model with interfacial layers between the NiFe and FeMn layers.

**Figure 4:**
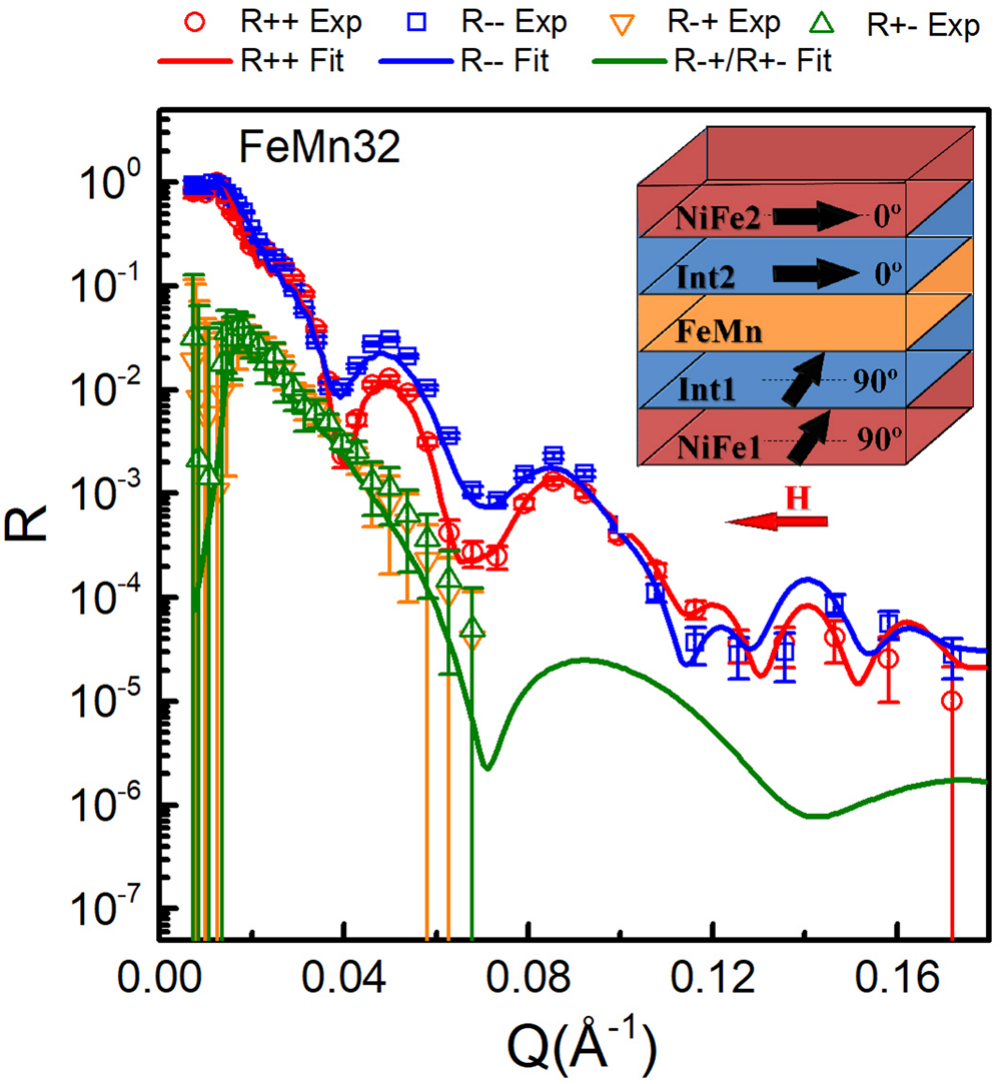
The PNR data and fits at *H* = −20 Oe for the FeMn32 sample. The magnetization models used in the fits are shown with arrows indicating the direction of the magnetization and with the angles of the magnetization with respect to the cooling field direction.

Figure 4 shows the R^++^, R^−−^, R^+−^ and R^−+^ reflectivity curves measured at an external field of −20 Oe for the FeMn32 sample which showed no difference between the minor and major hysteresis loops. Using the magnetization profile from the fitted PNR data at saturating fields, the direction of the magnetization of the magnetic layers at −20 Oe can be fitted from the SF reflectivity measurement. Notice that only the low *Q* SF reflectivities are measured, due to the large error bars of the SF signals in the high-*Q* range^31^, which arise from the low signal strength. We found that at −20 Oe the pinned-NiFe2 layer and the Int2 interfacial layer reverse back along the direction of the cooling field, in agreement with magnetometry, however the NiFe1 and Int1 layers rotate to nearly 90°. The error in the fitting parameters are estimated by a 5% increase over the optimum figure of merit FOM∼${\sum _{i-1}^{N}(\frac{{R}_{i}^{best}-{R}_{i}^{pertubed}}{{R}_{i}^{best}})}^{2}$^51^, where ${R}_{i}^{best}$ is the best-fitted reflectivities to the experimental results, ${R}_{i}^{pertubed}$ is the calculated reflectivities on the variance of a single fitting parameter, respectively. Note that the uncertainties in the magnetization orientation of the interfacial layers are relatively large compared to that of the NiFe layers. This is mainly due to the fact that the very thin interfacial layers possess relatively weak magnetization. However, the 90° difference between the Int1 and Int2 layers for FeMn32 sample does imply a strong perpendicular coupling between them.

**Figure 5:**
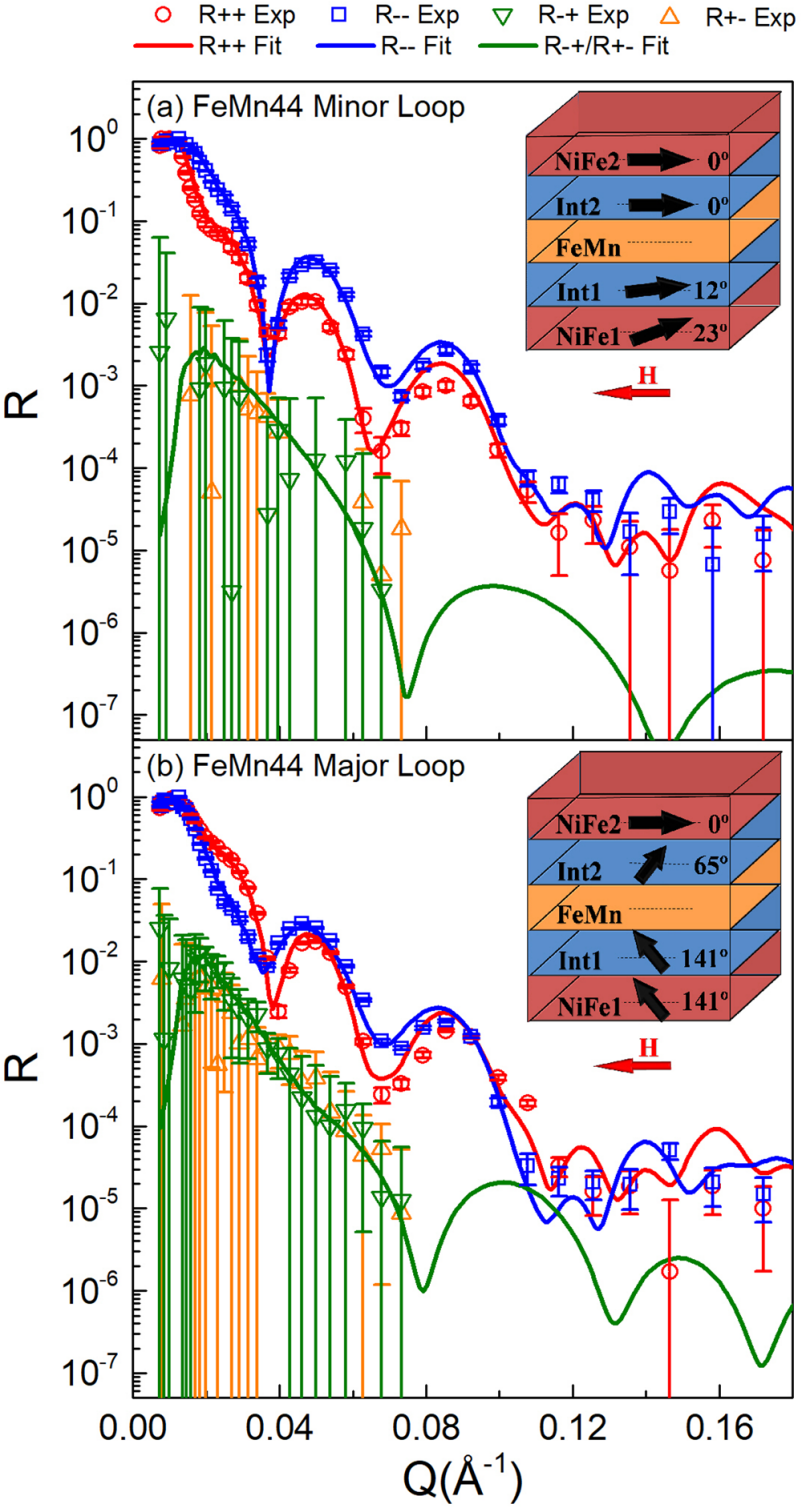
The PNR data and fits at *H* = −20 Oe for the FeMn44 sample during the major loop measurement (**a**) and the minor loop measurement (**b**). The magnetization models used in the fits are shown with arrows indicating the direction of the magnetization and with the angles of the magnetization with respect to the cooling field direction.

The FeMn44 sample showed a large separation in the major loop and the minor loop of the NiFe1 layer, therefore, two kinds of SF neutron reflectivity measurement were undertaken. Firstly, we set up the measuring field following the minor loop without reversing the pinned-NiFe2 layer, and then measured the NSF and SF reflectivity at *H* = −20 Oe. From the fitting results in Fig. 5(a), the NiFe1 and Int1 layers almost reverse back, consistent with the VSM results as shown in Fig. 1(c). The two NiFe layers have about 12° difference in magnetization orientation, suggesting that the 90° interlayer coupling is weaker. We then set up the measuring field following the major loop, and then measure the NSF and SF reflectivity at *H* = −20 Oe. The fit in Fig. 5(b), shows that the pinned-NiFe2 layer reverses back along the cooling field. However, the adjacent Int2 layer shows a non-collinear magnetization orientation with the pinned-NiFe2 layer. Meanwhile, the interfacial Int1 and Int2 layers have about 76° difference in orientation during the magnetic reversal. It suggests that a 90° coupling through the thick FeMn layer still exists, but becomes weak, compared with the EB effect at the NiFe1/FeMn interface. It should be noted that our PNR data are best fitted within a simple magnetization rotation model due to the fact that significant SF signals cannot be observed in alternative reversal models, e.g. the domain formation or the out-of-plane reversal model^31^. The neutron data did not show evidence of off-specular scattering which is consistent with the domain rotation model.

## Discussion

Several coupling mechanisms can give rise to the 90° interlayer coupling in FM/NM/FM trilayers. For example, to explain biquadratic coupling several extrinsic mechanisms are suggested by Demokritov^52^. In the case that the spacer is an AF layer, Slonczewski proposed a so-called *proximity magnetism* model to describe the perpendicular interlayer coupling, which has been developed for an uncompensated interface with sufficiently large lateral interface fluctuations^53^. Encouraged by the PNR data, we assume that there is a competition between the 90° interlayer coupling and the AF anisotropy. 90° interlayer coupling arises between the Int1 and Int2 layer, which decays with increasing thickness of the AF spacer layer. To further investigate the underlying mechanism, a Monte Carlo simulation was performed. The details of the simulation can be found in the supplementary materials. The simulated model consists of one FM layer (labeled FM1 layer) and a pinned-FM layer (labeled FM2 layer) separated by an AF spacer layer. The pinned-FM layer exchange couples to an extra AF layer (labeled AF2 layer). With increasing AF thickness, there is a weakening of the 90° interlayer coupling and an enhancement of the AF anisotropy. The weakening of the 90° interlayer coupling results from the increased distance between the FM layers, whilst the enhanced AF anisotropy may result from the effective expansion of the AF volume^54^, an improvement in the crystalline texture^39,40^, or an increase in the bulk inhomogeneities^37^. As the relative scale of both energy changes is unknown and may complicate the analysis without contributing to our physical understanding we simplify the modeling by fixing the thickness and the uniaxial anisotropy of the AF layer and only changing the strength of the interlayer coupling across the AF layer (see the appendix for further details on the calculation). In this way, the effect of the competition between 90° interlayer coupling and AF anisotropy can be qualitatively investigated.

**Figure 6:**
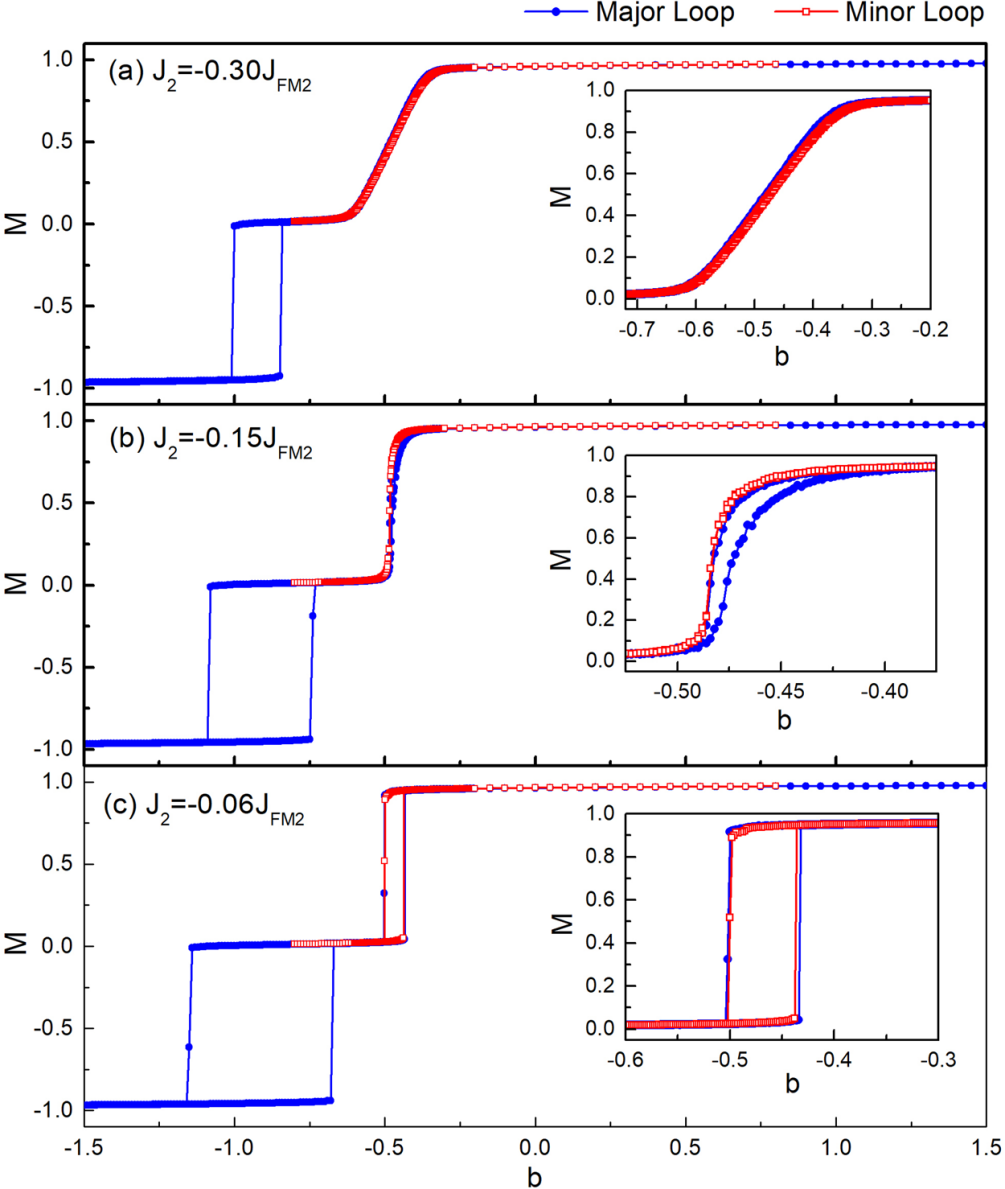
The hysteresis loops for FM1/AF/FM2/AF2 system with different interlayer biquadratic coupling strengths *J*_2_ = −0.30 *J*_FM2_ (**a**), −0.15 *J*_FM2_ (**b**) and −0.06 *J*_FM2_ (**c**). Red squares and blue circles refer to the minor loop and major loop measurements, respectively. The insets show the enlarged FM1 loops. The reduced magnetic field *b* and the normalized magnetization of the FM1 layer *M* are used.

Figure 6 shows the calculated hysteresis loops for the trilayer system with varying interlayer coupling. As shown in Fig. 6(a), the minor loop and major loop of the FM1 layer overlap with each other and are both tilted in shape in the presence of a strong 90° interlayer biquadratic coupling. By weakening the interlayer coupling to *J*_2_ = −0.15*J*_FM2_, both FM1 loops become much squarer in shape in good agreement with the experimental observations (Fig. 1). Moreover, with the ascending branch of the FM1 major loop shifting towards zero field, a pronounced difference between the minor loop and major loop is observed, indicating a clear EB transfer effect. Further reduction of the interlayer coupling causes the differences between the major and minor loops to vanish. As shown in Fig. 6(c), square loops are obtained for the FM1 layer showing an indiscernible difference between the major loop and minor loop with *J*_2_ = −0.06 *J*_FM2_. Notice that the presence of the EB field difference observed in Fig. 2 can be simply reproduced by setting appropriate relative strengths of the interlayer coupling and the AF anisotropy in the micromagnetic simulations. This simulation demonstrates that the competition between the two energy terms plays a crucial role in the EB transfer effect.

From these results we may draw some conclusions on the behavior in NiFe1/FeMn/pinned-NiFe2 multilayers. In FeMn32, 90° interlayer coupling dominates the competition since there is only a weak AF anisotropy, which causes the 90° difference in orientations of the Int1 and Int2 layers. Increasing the AF anisotropy in the FeMn44 sample driven by the increased thickness reduces the angle difference between the Int1 and Int2 layers. During the minor hysteresis loop, the Int2 and pinned-NiFe2 layers retain their previous orientation. However, when the Int2 and pinned-NiFe2 layers reverse along the major hysteresis loop, the reversed Int2 does not totally reverse back although the pinned-NiFe2 has reversed back. Thus, due to the interlayer coupling, Int1, and NiFe1 are all still in the direction of the external field, resulting in a small exchange bias field (*H*_EB2_). The role of the interlayer coupling appears like an energy barrier during the magnetization reversal. When the spacer is very thick, the interlayer coupling becomes so weak that the strong exchange coupling of FeMn/NiFe1 dominates the energy competition. Both interfacial layers follow the adjacent FM layers and become almost independent of each other. Thus, the whole magnetization configurations of the NiFe1/FeMn/pinned-NiFe2 system, which are determined by the competition between the interlayer coupling and the AF anisotropy, should be taken into account for a better understanding of the EB transfer effect.

## Conclusion

In conclusion, we have investigated the magnetic reversal behavior of NiFe/FeMn/pinned-NiFe multilayers with varying FeMn thicknesses by using PNR^55^. Based on the PNR analysis, 90° interlayer coupling through thick FeMn spacers beyond the spin fluctuation of AF has been identified, which may play a key role in the transfer of the EB effect from one NiFe/FeMn interface to another. Monte Carlo simulations of the layers confirm that the competition between the interlayer coupling energy and the AF anisotropy energy gives rise to the transfer of the EB effect. Our findings may shed light on the mechanism behind the transfer of exchange bias through antiferromagnets.

## Supplementary information

FeMn-sm
