# Supplementary material for: Probing the Transfer of the Exchange Bias Effect by Polarized Neutron Reflectometry
Source: Sci Rep. 2019 Apr 30;9:6708. doi: 10.1038/s41598-019-43251-1 (PMC6491425; doi:10.1038/s41598-019-43251-1)
Supplement: Supplementary file 1 — FeMn-sm [file 41598_2019_43251_MOESM1_ESM.pdf]

Supplementary Information for

Probing the Transfer of the Exchange Bias Effect by Polarized Neutron Reflectometry

X.Z. Zhan, G. Li, J.W. Cai, T. Zhu*, J.F.K. Cooper, C.J. Kinane, and S. Langridge

Correspondence to: tzhu@aphy.iphy.ac.cn (T.Z.)

I. The critical thickness of FeMn

The exchange bias blocking temperature, T_b, for an AF layer is the temperature at or above which the exchange bias effect was no longer present. In general, T_b was found to decrease with decreasing thickness of the AF. In other words, there is a critical thickness t_c, which the EB field vanishes at room temperature when T_b is below the room temperature. To determine t_c of FeMn we used, we prepared a series of samples, NiFe(100 Å)/FeMn(t Å)/Ta(30 Å). As shown in figure 1S, the EB vanishes when the thickness of FeMn is smaller than 24 Å.

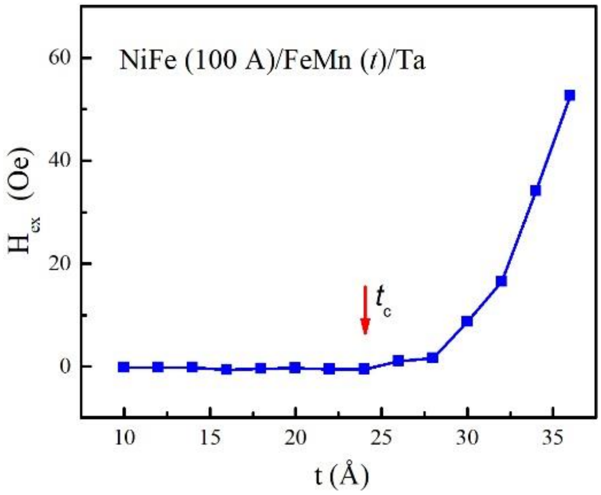

Fig. 1S The AF thickness dependence of exchange bias fields.

II. The PNR reflectivities

Fig. 2S shows the PNR data and fitting results for the FeMn44 sample. Similar to sample FeMn32, we also found a good agreement between our simulation and the experimental data when we added two interfacial layers at the NiFe/FeMn interfaces. Based on this result, we have done the spin-flip analysis using the PNR data at H = -20 Oe, which has been shown in Figure 5.

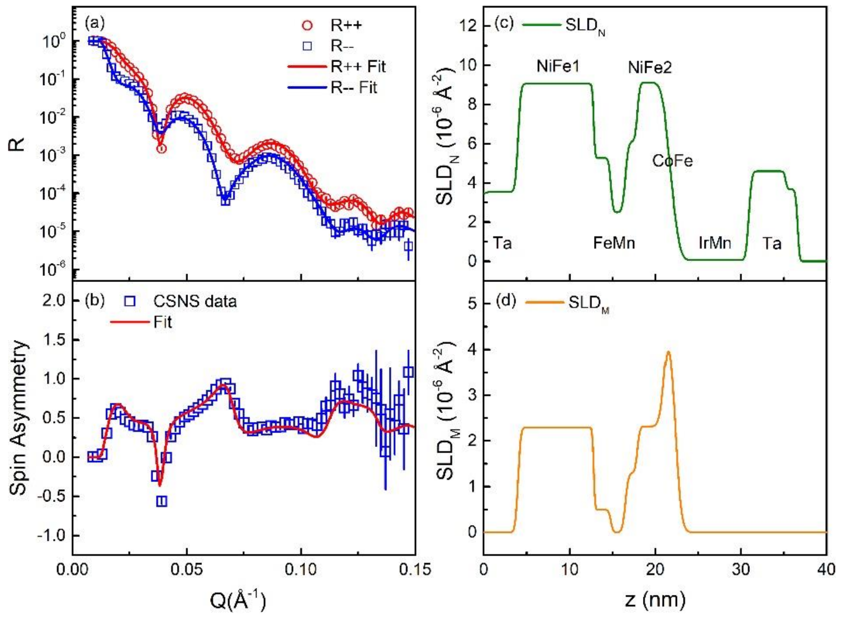

Figure 2S. The PNR analysis for the FeMn44 sample. (a) The NSF PNR reflectivities for the FeMn44 sample in the saturation state. (b) The spin asymmetry ratio obtained from the experimental and the calculated reflectivities. The nuclear SLD_N (c) and magnetization SLD_M (d) profiles for fitting models with interfacial layers. The solid lines present the best fits with the model with interfacial layers.

III. Monte Carlo simulation

The Monte Carlo model is constructed on a simple cubic lattice with a proper lateral extension of $L\times L$ (L=60) where periodic boundary conditions are applied[1]. In the longitude direction, the system consists of the FM1 monolayer, the AF layer with two

monolayers and the FM2 layer. The FM2 monolayer is exchange coupled to the AF2 layer to induced biased loops. All spins are modeled as classical Heisenberg spins $\vec{S}$ with the easy axis along the x axis and the nearest-neighbor exchange coupling constants J_{FM2}, $J_{\mathrm{FM1}}=J_{\mathrm{FM2}}/2$, $J_{\mathrm{AF}}=J_{\mathrm{AF2}}=-J_{\mathrm{FM2}}/5$ for the FM2 layer, the FM1 layer, and two AF layers, respectively[2]. To distinguish the hysteresis loop of the FM1 from that of FM2, different anisotropy constants $d_{\mathrm{x\_FM1}}= J_{\mathrm{FM2}}/5$ and $d_{\mathrm{x\_FM2}}= J_{\mathrm{FM2}}/2$ are used for the FM1 and FM2, respectively. A strong anisotropy $d_{\mathrm{x\_AF2}}=6J_{\mathrm{FM2}}$ is assumed in AF2 layer to induce a large EB field in the pinned-FM monolayer. The dipolar interactions are mimicked as an in-plane anisotropy $d_z=-0.1\,J_{\mathrm{FM2}}$ in all layers[2]. In addition, the interlayer coupling between the two FM layers contains the bilinear coupling and the biquadratic coupling with coupling constants J_1 and J_2 respectively[3,4]. Thus with an external field applying along the x axis, the Hamiltonian of the system can be written as,

$$
\begin{aligned}
H = &-\sum_{<i,j>} J\,\vec{S}_i\cdot\vec{S}_j - \sum_i (d_x S_{ix}^2 + d_z S_{iz}^2 + B S_{ix}) \\
&-J_{\mathrm{INT}}\left(\sum_{<i\in \mathrm{FM1},\, j\in \mathrm{AF}>} \vec{S}_i\cdot\vec{S}_j + \sum_{<i\in \mathrm{AF},\, j\in \mathrm{FM2}>} \vec{S}_i\cdot\vec{S}_j + \sum_{<i\in \mathrm{FM2},\, j\in \mathrm{AF2}>} \vec{S}_i\cdot\vec{S}_j \right) \\
&-J_1 \sum_{<i\in \mathrm{FM1},\, j\in \mathrm{FM2}>} \vec{S}_i\cdot\vec{S}_j - J_2 \sum_{<i\in \mathrm{FM1},\, j\in \mathrm{FM2}>} \left(\vec{S}_i\cdot\vec{S}_j\right)^2
\end{aligned}
$$

The first line gives the nearest-neighbor exchange coupling energy, the magnetocrystaline anisotropy energy, the in-plane anisotropy energy of all four layers and the Zeeman energy in the presence of an external field. Note that J and dx take different values for spins in different layers as stated above. The interfacial coupling energies are given on the second line with coupling constant $J_{\mathrm{INT}}=J_{\mathrm{FM2}}/2$ for all three different interfaces. The third line denotes the interlayer coupling between two FM layers with $J_1=0.01J_{\mathrm{FM2}}$ and varied J_2. To simply model the competition between the interlayer coupling and the AF anisotropy energy, a strong AF anisotropy constant

d_{x_AF}=6.5J_{FM2} is fixed in the AF layer and the biquadratic coupling is weaken gradually to mimic the effect of increasing thickness of the AF layer in experiments, *i.e.*, J_2=-0.30J_{FM2}, -0.15J_{FM2}, -0.06J_{FM2}. $\vec{S}_i$ is assumed to be a unit vector to simplify the calculations. The reduced magnetic field $b=B/J_{FM2}$ and the normalized magnetization of the FM1 layer $M = \frac{\sum_{<i \in FM1>} S_{ix}}{L^2}$ are used in the simulations. The field-cooled hysteresis loop of the system is obtained with the heat-bath algorithm. We have checked the situations with different lateral extension L to make sure that no related finite-size effect is included in the simulations.

1. Hu, Y. *et al.* Exchange bias and its propagation in ferromagnetic/ antiferromagnetic/ferromagnetic trilayers. *J. Appl. Phys.* **114**, 153901 (2013).
2. Usadel, K. D. & Nowak, U. Exchange bias for a ferromagnetic film coupled to a spin glass. *Phys. Rev. B* **80**, 014418 (2009).
3. Slonczewski, J. C. Overview of Interlayer Exchange Theory. *J. Magn. Magn. Mater.* **150**, 13-24 (1995).
4. Demokritov, S. O. Biquadratic interlayer coupling in layered magnetic systems. *J. Phys. D: Appl. Phys.* **31**, 925-941 (1998).
